# M2 Muscarinic Receptor Stimulation Induces Autophagy in Human Glioblastoma Cancer Stem Cells via mTOR Complex-1 Inhibition

**DOI:** 10.3390/cancers16010025

**Published:** 2023-12-20

**Authors:** Claudia Guerriero, Marianna Manfredelli, Carlo Matera, Angela Iuzzolino, Luciano Conti, Clelia Dallanoce, Marco De Amici, Daniela Trisciuoglio, Ada Maria Tata

**Affiliations:** 1 Department of Biology and Biotechnologies Charles Darwin, Sapienza University of Rome, 00185 Rome, Italy; claudia.guerriero@uniroma1.it (C.G.); manfredellimarianna@gmail.com (M.M.); angela.iuzzolino@uniroma1.it (A.I.); 2 Department of Pharmaceutical Sciences, University of Milan, 20133 Milan, Italy; carlo.matera@unimi.it (C.M.); clelia.dallanoce@unimi.it (C.D.); marco.deamici@unimi.it (M.D.A.); 3 Institute of Molecular Biology and Pathology, National Research Council, 00185 Rome, Italy; daniela.trisciuoglio@uniroma1.it; 4 Department of Cellular, Computational and Integrative Biology—CIBIO, University of Trento, 38123 Trento, Italy; luciano.conti@unitn.it; 5 Research Centre of Neurobiology Daniel Bovet, Sapienza University of Rome, 00185 Rome, Italy; 6 Consortium Interuniversity Biotechnologies (CIB), University of Ferrara, 44121 Ferrara, Italy

**Keywords:** glioblastoma, cancer stem cells, M2 muscarinic receptor, orthosteric and dualsteric muscarinic agonism, autophagy, apoptosis, mTORC1

## Abstract

**Simple Summary:**

Tumor cells use autophagy as a pro-survival strategy. However, several studies have shown that excessive stimulation of the autophagic process can promote cell death; in this context, autophagy acquires an antitumor effect. To further explore the consequences of the cytotoxic effects induced by M2 muscarinic receptor activation that we have previously described both in glioblastoma multiforme (GBM) stable cell lines and in human GBM cancer stem cells, here we investigated the involvement of autophagy and apoptosis in the cell death engendered by treatment with M2 muscarinic agonists. Moreover, we compared the effects mediated by orthosteric and dualsteric M2 muscarinic agonists in the modulation of these different mechanisms of cell death.

**Abstract:**

Background: Although autophagy is a pro-survival process of tumor cells, it can stimulate cell death in particular conditions and when differently regulated by specific signals. We previously demonstrated that the selective stimulation of the M2 muscarinic receptor subtype (mAChR) negatively controls cell proliferation and survival and causes oxidative stress and cytotoxic and genotoxic effects in both GBM cell lines and GBM stem cells (GSCs). In this work, we have evaluated whether autophagy was induced as a downstream mechanism of the observed cytotoxic processes induced by M2 mAChR activation by the orthosteric agonist APE or the dualsteric agonist N8-Iper (N8). Methods: To assess the activation of autophagy, we analyzed the expression of LC3B using Western blot analysis and in LC3B-EGFP transfected cell lines. Apoptosis was assessed by measuring the protein expression of Caspases 3 and 9. Results: Our data indicate that activation of M2 mAChR by N8 promotes autophagy in both U251 and GB7 cell lines as suggested by the LC3B-II expression level and analysis of the transfected cells by fluorescence microscopy. Autophagy induction by M2 mAChRs is regulated by the decreased activity of the PI3K/AKT/mTORC1 pathway and upregulated by pAMPK expression. Downstream of autophagy activation, an increase in apoptosis was also observed in both cell lines after treatment with the two M2 agonists. Conclusions: N8 treatment causes autophagy via pAMPK upregulation, followed by apoptosis in both investigated cell lines. In contrast, the absence of autophagy in APE-treated GSC cells seems to indicate that cell death could be triggered by mechanisms alternative to those observed for N8.

## 1. Introduction

Autophagy is an evolutionarily conserved catabolic process in all eukaryotes, from yeast to humans. In normal conditions, autophagy is necessary to maintain cellular homeostasis through the degradation of dysfunctional organelles, protein aggregates, or misfolded proteins that are delivered to the lysosomes for recycling [1]. One of the classes of autophagic pathways consists of macro-autophagy [2], involving the sequestration of substrates within double-membrane cytosolic vesicles called “autophagosomes”. These organelles fuse with lysosomes, generating a single-membrane organelle known as an “autolysosome”, which represents the degradative compartment of the entire process [3,4]. Two main factors are involved in the regulation of the autophagic process, i.e., the TOR complex-1 (TORC1) and the AMP-activated protein kinase (AMPK), which play opposite roles. Indeed, TORC1 negatively regulates autophagy, both in *S. cerevisiae* and in mammals [5]. Conversely, AMPK inhibits the TORC1 pathway through the phosphorylation of both its inhibitor protein TSC2 (tuberin) [6] and Raptor, a further TORC1 component [7]. In addition, AMPK activates the Unc-5-like kinase 1 (ULK1), the serine/threonine protein kinase that triggers autophagy [8].

Today, the role of autophagy in tumors remains a controversial issue. While numerous studies have shown that autophagy promotes tumor suppression in the early stage of tumorigenesis and autophagy-associated death of cancer cells, other reports have highlighted the role of autophagy as a pro-tumor-survival process under conditions of environmental stress [9,10].

Glioblastoma multiforme (GBM), the most lethal human brain tumor, has lower levels of protein markers of autophagy than low-grade astrocytic tumors [11,12]; in particular, ULK1 and ULK2 levels are significantly lower in GBM patients than in healthy controls [13]. An analysis conducted according to the Karnofsky Performance Scale index, which allows the classification of patients based on their functional impairment, reported that a high expression of Microtubule-associated protein 1A/1B-light chain 3 (LC3), a hallmark of autophagy, was correlated with an improved patient prognosis [14].

In addition, hyper-phosphorylation of AKT and mTOR has been detected in patients with grade-III and grade-IV gliomas. These factors, when active, are associated with the downregulation of the autophagic process and increased cell proliferation and stemness of glioma stem cells [15,16]. Considering this evidence on the role of autophagy as a tumor suppressor, drugs with synergistic action with the chemotherapy agents already in use, notably temozolomide, are being developed [10].

For many years, our group has been studying the effects of M2 muscarinic acetylcholine receptor (M2 mAChR) activation in GBM. mAChRs are G protein-coupled receptors (GPCRs) involved in the regulation of several fundamental processes in both central and peripheral nervous systems [17]. Moreover, the expression of mAChRs in different tumors and their strategic role in the modulation of cancer cell proliferation, survival and migration have been demonstrated [18,19,20,21,22]. In this framework, we have demonstrated that the activation of M2 mAChR by the orthosteric M2 agonist Arecaidine Propargyl Ester (APE) has negative effects on cell proliferation and survival, both in GBM cell lines and in GBM cancer stem cells (GSCs) [23,24]. Recently, we have also shown that treatment with an M2 agonist results in an increase in aberrant mitosis in U251 and U87 cell lines, caused by an altered mitotic spindle formation as a result of a reduction in the ability of microtubules to bind chromosomes. GBM cell lines are unable to proliferate after APE treatment, due to the triggering of catastrophic mitotic events and apoptosis [25].

To improve the therapeutic potential of mAChR ligands in different pathological conditions such as Alzheimer’s disease [26,27], schizophrenia [17,28] and cancer [21,23,29], novel hybrid molecules, named “dualsteric or bitopic agonists”, were designed, synthesized and tested. This kind of ligand, which has been reported also for various GPCR receptor families, simultaneously recognizes the acetylcholine (orthosteric) binding site and an additional, allosteric site. Such a cooperative interaction may increase the degree of selectivity among the subtypes and also promote signaling bias, since binding to the allosteric site may favor a functionally relevant receptor structure that controls a specific signaling pathway [30,31].

Iper-8-naphthalimide (N-8-Iper, N8) is a synthetic dualsteric agonist for the M2 mAChR previously characterized by our research group [31,32,33]. We demonstrated that N8 reduced GSC proliferation in a comparable manner to APE in two GSC lines, GB7 cells and G166 cells. Quite interestingly, N8 is active at doses (3 μM for GB7 cells and 25 μM for G166 cells) that are significantly lower than that of APE (100 μM) [32,33].

Starting from our previous results, here we investigated the downstream molecular targets linked to the cytotoxic effects observed after M2 mAChR activation with APE and N8 [23,33,34]. Moreover, we analyzed the signal transduction pathways involved in these processes, which could be affected by the different recognition modes of the two investigated muscarinic agonists.

We detected an upregulation of the autophagy process both in the U251 cell line and in GB7 cells after N8 treatment at high and low doses, while APE triggered autophagy only in U251 cells. Moreover, we studied the downregulation of the phosphoinositide 3-kinase (PI3K)/AKT/TORC1 pathway and the upregulation of AMPKα expression, which have been identified as two of the main pathways involved in the modulation of autophagy flux. Finally, the increased autophagy after M2 stimulation by N8 positively improved the apoptotic process, as shown by the increase in the cleaved form of Caspases 9 and 3.

## 2. Materials and Methods

### 2.1. Cell Cultures

The GSCs GB7 cells were obtained from a human GBM biopsy [35,36]. The cells were cultured on laminin-coated dishes (1 μg/mL; Sigma-Aldrich, St. Louis, MO, USA) and maintained in serum-free medium consisting of DMEM/F12 (Sigma-Aldrich, St. Louis, MO, USA) and Neurobasal medium (Gibco, Thermo Fisher Scientific, Waltham, MA, USA) (1:1; *v*:*v*) supplemented with 1% streptomycin, 50 IU/mL penicillin (Sigma-Aldrich, St. Louis, MO, USA), 1% glutamine (Sigma-Aldrich, St. Louis, MO, USA), 1% N2 supplement (Gibco, Thermo Fisher Scientific, Waltham, MA, USA), 2% B27 (Gibco, Thermo Fisher Scientific, Waltham, MA, USA), 20 ng/mL EGF (Recombinant Human Epidermal growth factor, Peprotec, London, UK) and 20 ng/mL FGF (Recombinant Human FGF-basic, ABM, Richmond, Canada). The GB7 cell culture was maintained at 37 °C in an atmosphere of 5% CO_2_/95% air. The human glioblastoma U251MG cell line was cultured in DMEM (Sigma-Aldrich, St. Louis, MO, USA) containing 10% fetal bovine serum (Sigma-Aldrich, St. Louis, MO, USA), 50 μg/mL streptomycin, 50 IU/mL penicillin, 2 mM glutamine (Sigma-Aldrich, St. Louis, MO, USA) and 1% non-essential amino acids (Sigma-Aldrich, St. Louis, MO, USA) and maintained at 37 °C in a 10% CO_2_ atmosphere.

### 2.2. Pharmacological Treatments

Arecaidine Propargyl Ester hydrochloride (APE, Sigma-Aldrich, Milan, Italy) is a synthetic compound obtained from the modification of arecaidine, a natural alkaloid derived from areca nut. M2 agonist APE was used to selectively stimulate the M2 mAChR subtype. The ability of this agonist to bind the M2 mAChR subtype was previously demonstrated in GBM stable cell lines (U87 and U251 cell lines) and in GSCs (GB7 and GB8 cells) by pharmacological binding experiments and knockdown of the receptors using an siRNA transfection pool [23,24]. Iper-8-naphthalimide (N8) was synthesized according to a known literature procedure [37]. Its ability to selectively bind the M2 mAChR subtype has been shown by pharmacological binding and M2 mAChR knockdown experiments [33]. To compare the activity of the two M2 receptor ligands, we first used both agonists at high concentrations (100 μM) and then adopted the lowest doses of N8 (25 μM and 3 μM) producing significant effects in the U251 cell line and GB7 cells, as shown previously [32].

### 2.3. U251 Cell Line Transfection and Immunofluorescence Analysis

Transfection was performed on the U251 cell line using the following expression vectors: EGFP-LC3B and mRFP-EGFP-LC3B. Cells were seeded on 6-well plates at the density of 2 × 10^5^/well and transfected at 80% confluence using Lipofectamine^TM^ 3000 Reagent (Invitrogen, Waltham, MA, USA), according to the manufacturer’s protocol. Forty-eight hours after transfection, the cells were maintained in a complete medium supplemented with geneticine (800 μg/mL; Sigma-Aldrich, St. Louis, MO, USA) to select only antibiotic-resistant clones containing the EGFP-LC3B fusion protein or mRFP-EGFP-LC3B fusion protein.

Stable U251-EGFP-LC3 or U251-mRFP-EGFP-LC3 cells were plated on coverslips arranged in 24-well plates at the density of 2 × 10^4^ cells. Cells were treated with 100 μM APE, 100 μM N8 and 25 μM N8 with or without 25 μM Chloroquine (CQ) for 72 h. Then, cells were washed 3 times with PBS and fixed with 4% paraformaldehyde in PBS for 20 min at room temperature. After 3 washes in PBS, cells were incubated with Hoechst 33,342 (1:1000, Sigma-Aldrich, St. Louis, MO, USA) for 10 min at room temperature (RT), for nuclei counterstaining, and then washed 3 times with PBS. At the end, coverslips were fixed on microscope slides with a PBS-glycerol (3:1; *v*/*v*) solution. The images were acquired with an Apotome Zeiss fluorescence microscope using Zeiss Zen lite software 3 (Zeiss, Oberkochen, Germany).

### 2.4. Cell Viability Assay

U251 cells were seeded on 96-well plates at the density of 1.5 × 10^4^ cells/well. After 24 h, cells were treated with N8 at different times (ranging from 24 to 96 h). Cell proliferation was evaluated using a colorimetric assay based on 3-(4,5-dimethylthiazol-2-y1)-2,5-diphenyltetrazolium bromide (MTT, Sigma-Aldrich, St. Louis, MO, USA) metabolization. The MTT assay was performed according to the protocol optimized by Mosmann [38]. MTT was dissolved in PBS at 5 mg/mL. The MTT stock solution (10×) was added and diluted (1×) in each well and then incubated at 37 °C for 3 h. Isopropanol (+0.04 M HCl + 1% Triton X-100) was used to dissolve the dark blue crystals. For each well, the optical density (OD) at 570 nm was measured using a Multiskan FC (Thermofisher Scientific, Waltham, MA, USA).

### 2.5. Protein Extraction and Western Blot Analysis

Cells were harvested in lysis buffer (Tris-EDTA 10 mM, 0.5% NP40, NaCl 150 mM), containing a protease inhibitor, and boiled for 5 min at 90 °C. The protein extracts were run on SDS-polyacrylamide gel (SDS-PAGE) and transferred to Polyvinylidene Difluoride (PVDF) sheets (Merck Millipore, Darmstadt, Germany). Membranes were blocked for 40 min in 5% non-fat milk powder (Sigma-Aldrich, St. Louis, MO, USA) in PBS containing 0.1% Tween-20 (PBS-Tween) and then incubated overnight at 4 °C with one of the following primary antibodies: anti-LC3B (1:1500, Sigma-Aldrich, St. Louis, MO, USA), anti-PI3 Kinase p85 (dilution 1:800, Cell Signaling, Danvers, MA, USA), anti-Phospho AKT^Thr308^ (1:800, Cell Signaling, Danvers, MA, USA), anti-AKT pan (1:800, Cell Signaling, Danvers, MA, USA), anti-Phospho AMPK*α*1/2^Thr172^ (dilution 1:800, Cell Signaling, Danvers, MA, USA), anti-AMPK*α*1 (dilution 1:1000, Immunological Science, Milan, Italy), anti-Phospho p70 S6 Kinase^Thr389^ (dilution 1:600, Immunological Science, Milan, Italy), anti-p70S6 Kinase (dilution 1:1000, Immunological Science, Milan, Italy), anti-Caspase 9 (dilution 1:2000, Immunological Science, Milan, Italy), anti-Caspase 3 (dilution 1:2000, Immunological Science, Milan, Italy), anti-β-Actin (1:2000, Immunological Science, Milan, Italy). β-Actin was used as a reference protein for loading control.

### 2.6. Statistical Analysis

Data are presented as the mean ± SEM. Statistical analysis was performed using one-way ANOVA followed by a Dunnett multiple comparison post-test. Data were considered statistically significant at * *p* < 0.05, ** *p* < 0.01 and *** *p* < 0.001. Data analyses were performed with GraphPad Prism 8 (GraphPad Software, La Jolla, CA, USA).

## 3. Results

### 3.1. Analysis of Autophagy in U251 Cell Line and GB7 Cells

**Figure 1:**
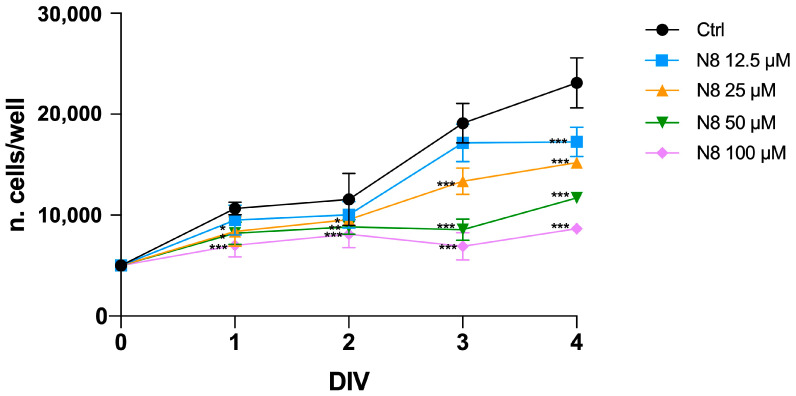
Effect of N8 (12.5 μM, 25 μM, 50 μM and 100 μM) on U251 cell growth at different time points of treatment (ranging from 1 to 4 days in vitro, DIV). Data represent the mean (±SEM) of four different experiments performed in sextuplicate. ANOVA test was used, followed by Dunnett’s post-test (N8 treated cells vs. untreated cells (Ctrl); *** *p* < 0.001; ** *p* < 0.01; * *p* < 0.05).

We demonstrated that selective activation of M2 mAChR by the dualsteric agonist N8 for G166 and GB7 GSCs causes a reduction in cell proliferation in a time- and dose-dependent manner [32,33]. For GB7 cells, the lowest concentration of N8 that produced a significant reduction in cell number was 3 μM [33]. Using the MTT assay, we evaluated the action of different concentrations of N8 after 24, 48, 72 and 96 h of treatment on the U251 cell line. As shown in Figure 1, the first dose of N8 capable of significantly reducing U251 cell proliferation was 25 μM. The lowest doses analyzed, 12.5 μM, 6 μM and 3 μM, did not alter cell proliferation compared to untreated cells until 72 h. For this reason, a 25 μM dose was used as the first effective dose in the U251 cell line for subsequent experiments.

**Figure 2:**
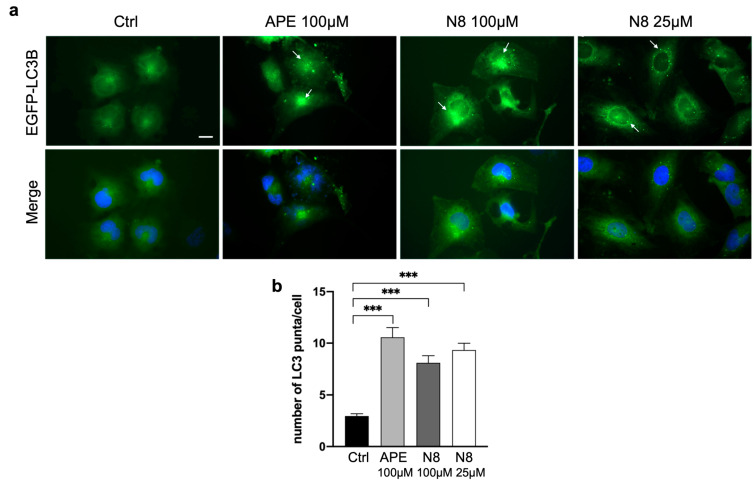
(**a**) Representative images of fluorescence microscopy analysis of the U251 cell line transfected with the EGFP-LC3B expression vector, treated for 72 h with 100 μM APE, 100 μM N8 or 25 μM N8. Nuclei were stained with Hoechst 33342. White arrows indicate green spots. Scale bar = 10 μm. (**b**) The quantification was performed by counting the number of green spots within each cell in 13 photographic fields for each experimental condition. All the experiments were performed in triplicate. The values (mean ± SEM) are reported as number of green dots/cells. For each experimental condition, about 150 cells were analyzed. ANOVA test was used, followed by Dunnett’s post-test (untreated cells (Ctrl) vs. M2 agonist-treated cells; *** *p* < 0.001).

To evaluate whether the M2 mAChR activation was able to induce autophagy, we analyzed the LC3 protein localization. LC3 is synthesized in its inactive form (pro-LC3) and subsequently converted to its active cytosolic form (LC3-I). LC3-I undergoes proteolytic cleavage at the C-terminus and is then converted to LC3-II by conjugation with phosphatidylethanolamine on the surface of nascent autophagosomes. The lipidated LC3 (LC3-II) is associated with autophagosomes and autolysosomes [39,40]. To observe and quantify the accumulation of LC3-II in autophagosomes, we stably transfected the U251 cells with the vector encoding the EGFP-LC3B fusion protein. Stably transfected cells were treated with 100 μM APE, 100 μM and 25 μM N8 for 72 h (Figure 2).

In fluorescence microscopy analysis (Figure 2a), green spots were observed in cells treated with M2 agonists, indicative of LC3 recruitment to the membrane of autophagosomes. In contrast, untreated cells showed predominantly uniformly scattered green fluorescence in the cells. A quantitative analysis was conducted by counting the green spots in each cell under the different treatment conditions. As shown in the graph in Figure 2b, a significant increase in the green spots present within the cells was observed after 72 h of 100 μM APE and 100 μM or 25 μM N8 treatments compared with untreated cells, suggesting a significant increase in the autophagy process after treatment with both M2 agonists.

To determine whether the accumulation of LC3B-II was the result of an increased de novo biosynthesis of autophagosomes or a blockage of the autophagy process, we analyzed LC3B protein turnover using fluorescence analysis in cells transfected with a pH-sensitive vector with double fluorescence (mRFP-EGFP-LC3B) and using Western blot analysis.

**Figure 3:**
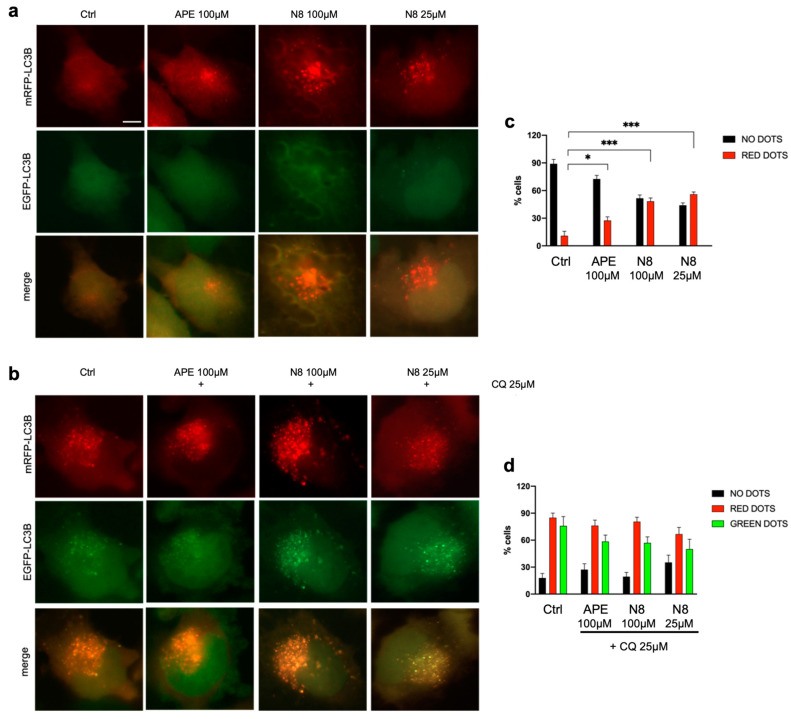
Representative images of fluorescence microscopy analysis of the U251 cell line stably transfected with the mRFP-GFP-LC3B expression vector, treated for 72 h with 100 μM APE, 100 μM N8 or 25 μM N8 in (**a**) the absence or (**b**) presence of 25 μM CQ. Scale bar = 5 μm. (**c**,**d**) The quantification was performed by counting the number of cells without dots (black bars), with green dots or with red dots in 15 photographic fields for each experimental condition performed in triplicate. The values (mean ± SEM) are reported as a percentage of red or green positive cells with respect to total active cells. For each experimental condition, about 100 cells were analyzed. The ANOVA test was used to evaluate the statistical significance of group differences, followed by Dunnett’s Multiple Comparison Test to identify the statistical significance between pairs, Ctrl (untreated cells) vs. M2 agonist-treated cells (*** *p* < 0.001, * *p* < 0.05). In (**d**), no significant difference was observed between red and green dots for each experimental condition.

The mRFP-EGFP-LC3B vector allowed us to study the progression from autophagosomes to autolysosomes, exploiting the different pH levels of the two organelles and the difference in the pH sensitivity of EGFP, which is sensitive to acidic pH, and RFP, which is resistant to pH changes. The U251 cell line was stably transfected with the mRFP-EGFP-LC3B vector, and then cells were treated with 100 μM APE and 100 μM N8 or 25 μM N8 for 72 h (Figure 3). In this experiment, the LC3B localization was analyzed in the presence or absence of 25 μM Chloroquine (CQ), which is an inhibitor of late-stage autophagy as it blocks the fusion of autophagosomes to lysosomes [41]. The U251-mRFP-EGFP-LC3B cells showed an increase in red fluorescence spots and the absence of green ones, signs of the formation of autophagolysosomes evidenced by the persistence of red fluorescence and by the absence of green fluorescence, which can be detected only in an acidic pH environment (Figure 3a). The images in Figure 3a,b are high-magnification images of representative fields of the different experimental conditions. Fluorescence images at lower magnification, showing the same effect of Chloroquine, are shown in Supplementary Figure S1. Quantitative analysis performed by counting all the fluorescent cells, those with only red spots and those with green spots showed that after treatment with the M2 mAChR agonists, a significant increase in the percentage of cells with only red spots was observed. The absence of green spots indicates that the M2 agonists trigger the autophagy process and allow a proper fusion between autophagosomes and lysosomes (Figure 3c). After CQ treatment, an increase in green spots was observed in the cells, indicative of the autophagy process being blocked at the autophagosome stage, where neutral pH allows the mRFP-EGFP-LC3B construct to express both green and red fluorescence (Figure 3b). However, quantitative analysis did not reveal any significant modulation in the percentage of cells with green spots between the different experimental conditions (Figure 3d).

**Figure 4:**
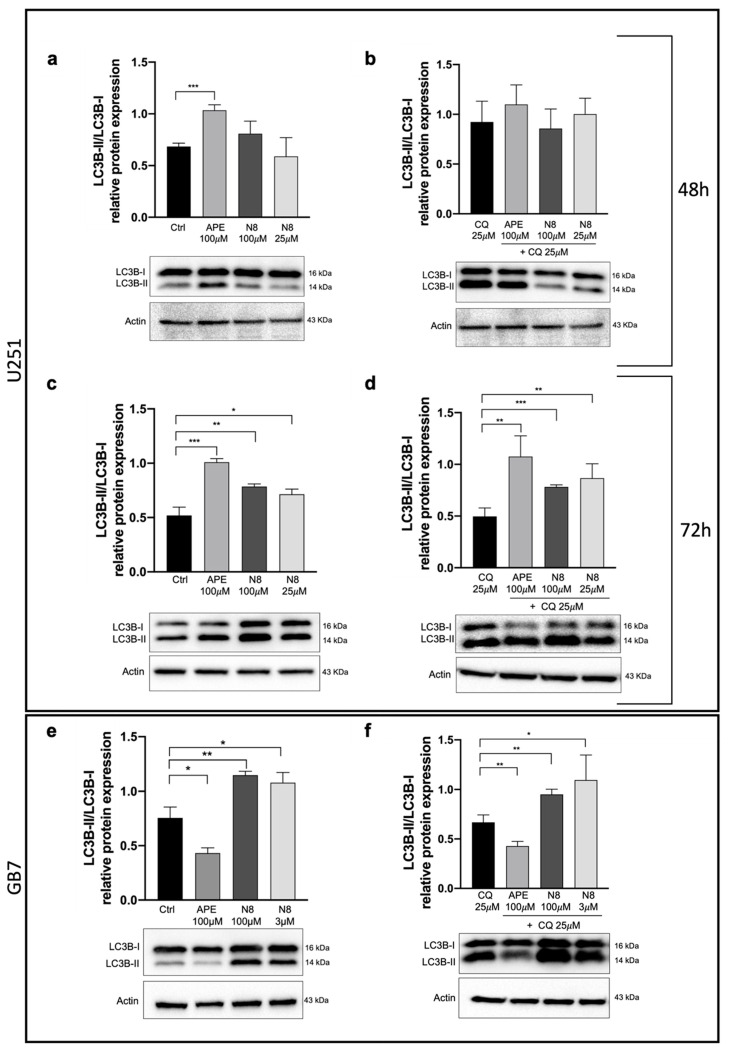
Representative Western blot relative to LC3B-II/I expression after 48 h (**a**) and 72 h (**c**) of treatment with 100 μM APE, 100 μM N8 or 25 μM N8 and after 48 h (**b**) and 72 h (**d**) of co-treatment with 25 μM CQ and APE (100 μM) or N8 (100 μM and 25 μM) + CQ in the U251 cell line. (**e**) Representative Western blot analysis of LC3-II/I expression after 48 h of treatment with 100 μM APE, 100 μM N8 or 3 μM N8 and (**f**) after 48 h of co-treatment with 25 μM CQ and APE (100 μM) or N8 (100 μM and 3 μM) + CQ in GB7 cells. Actin was used as an internal reference protein. The graphs show the densitometric analysis of the bands of the Western blot analysis for LC3B-II normalized with the bands of LC3B-I protein. The data are the average (mean ± SEM) of three independent experiments. The ANOVA test was used to evaluate the statistical significance of group differences, followed by Dunnett’s Multiple Comparison Test to identify the statistical significance between pairs, Ctrl (untreated cells) vs. M2 agonist-treated cells (*** *p* < 0.001, ** *p* < 0.01, * *p* < 0.05). The uncropped blots are shown in Supplementary Materials.

Then, we investigated the LC3B protein expression using Western blot analysis, not only in the U251 cell line but also extending the analysis to the previously characterized GSCs, GB7 cells [33]. Using Western blot analysis, we quantified the expression of LC3B-I and LC3B-II forms in both cell lines after treatment with M2 agonists. A 3 μM treatment with N8 was used in GB7 cells since this was the lowest effective dose in reducing cell proliferation [32]. Cells were also treated in combination with 25 μM CQ to study the progression of autophagy flux in the presence or in the absence of an autophagy inhibitor. In the U251 cell line, after 48 h, an increase in the LC3B-II form compared with LC3B-I was observed only with APE treatment (Figure 4a). Enhancement of the LC3B-II form, after treatment with N8 at high (100 μM) and low (25 μM) doses, became evident only after 72 h of treatment, when the increase in the LC3B-II/LC3B-I ratio in APE-treated cells was still meaningful (Figure 4c). After 72 h of treatment, the increased LC3B-II/LC3B-I ratio was also observed in U251 cells, in which M2 agonists were provided in combination with CQ compared with CQ alone (Figure 4d); on the other hand, no significant change in this ratio was revealed under the same experimental conditions after treatment for 48 h (Figure 4b). As shown in Figure 4e, in GB7 cells, treatment with 100 μM APE for 48 h did not overexpress LC3B-II compared with untreated cells, whereas in N8-treated cells, there was an upregulation of LC3B-II compared with LC3B-I at both high (100 μM) and low (3 μM) doses. Even prolonged APE treatment (72 h) did not cause overexpression of LC3B-II, and no additional increase was observed after N8 treatment. Conversely, a significant increase in LC3B-II expression was measured after 48 h of treatment with the combination of N8 (100 μM or 3 μM) and CQ, compared with cells treated with 25 μM CQ alone (Figure 4f). The increased accumulation of autophagosomes observed in the presence of CQ after stimulation of the M2 receptor with N8 suggests the role of this receptor in triggering the autophagy process in GB7 cells.

### 3.2. Analysis of TORC1 and AMPK Expression in U251 Cell Line and GB7 Cells

**Figure 5:**
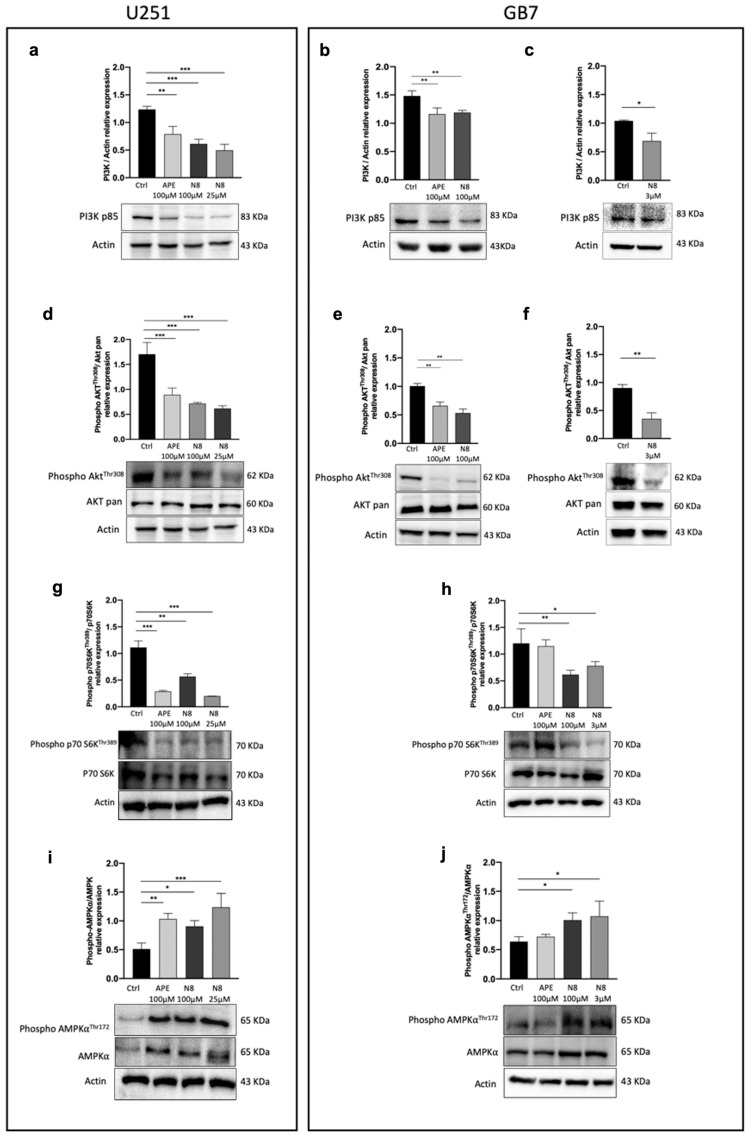
(**a**) Representative Western blot analysis for PI3K p85 expression after 72 h of treatment with 100 μM APE, 100 μM N8 or 25 μM N8 in the U251 cell line and (**b**) after 72 h of treatment with 100 μM APE, 100 μM N8 and (**c**) 3 μM N8 in GB7 cells. The graphs show the densitometric analysis of the bands of Western blot analysis for PI3K p85 normalized with the bands of actin protein. (**d**) Representative Western blot analysis of the expression of AKT phosphorylated at Thr 308 (Phospho-AKT^Thr308^) after 72 h of treatment with 100 μM APE, 100 μM N8 or 25 μM N8 in the U251 cell line and (**e**) after 72 h of treatment with 100 μM APE, 100 μM N8 and (**f**) 3 μM N8 in GB7 cells. The graphs show the densitometric analysis of the bands of Western blot analysis for Phospho-AKT^Thr308^ normalized with the bands of AKT pan. Actin was used as a reference protein. (**g**) Representative Western blot analysis of the expression of p70 S6K phosphorylated at Thr 389 (Phospho-p70 S6K^Thr389^) after 72 h of treatment with 100 μM APE, 100 μM N8 or 25 μM N8 in the U251 cell line and (**h**) after 72 h of treatment with 100 μM APE, 100 μM N8 and 3 μM N8 in GB7 cells. The graphs show the densitometric analysis of the bands of Western blot analysis for Phospho-p70 S6K^Thr389^ normalized with the bands of p70 S6K. Actin was used as a reference protein. (**i**) Representative Western blot analysis of the expression of AMPK*α* phosphorylated at Thr 172 (Phospho-AMPK*α*^Thr172^) after 72 h of treatment with 100 μM APE, 100 μM N8 or 25 μM N8 in the U251 cell line and (**j**) after 72 h of treatment in GB7 cells. The graphs show the densitometric analysis of the bands of Western blot analysis for Phospho-AMPK*α*^Thr172^ normalized with the bands of AMPK*α*. Actin was used as a reference protein. The data are the average (mean ± SEM) of three independent experiments. The ANOVA test was used to evaluate the statistical significance of group differences, followed by Dunnett’s Multiple Comparison Test to identify the statistical significance between pairs, Ctrl (untreated cells) vs. M2 agonist-treated cells (*** *p* < 0.001, ** *p* < 0.01, * *p* < 0.05). The uncropped blots are shown in Supplementary Materials.

With the aim of evaluating the molecular mechanism involved in the M2 receptor-triggered autophagy, we assessed the expression of proteins implicated in the PI3K/AKT pathway and of the two regulators of the catabolic process, AMPK and TORC1. Thus, using Western blot analysis, we investigated the PI3K/AKT/mTOR signaling pathway upon APE or N8 treatments in both the U251 cell line and GB7 cells. We examined the expression of PI3K p85 and AKT, phosphorylated at Threonine 308 (Thr 308), which is activated by *Phosphoinositide-dependent kinase-1* (PDK1), which in turn is activated by PI3K. As shown in Figure 5a, in the U251 cell line, PI3K p85 is downregulated after 72 h of treatment with both 100 μM APE and N8 at high (100 μM) and low (25 μM) doses. M2 agonists were able to downregulate the PI3K p85 protein even in the GB7 cells after 72 h of treatments (Figure 5b,c). The same trend was observed for the expression of the active form of the AKT protein, phosphorylated at Thr 308; in fact, APE and N8 caused a significant downregulation of Phopho-AKT (Thr 308) expression, compared to AKT (pan) which does not discriminate between the active and inactive forms, in both the U251 cell line (Figure 5d) and GB7 cells (Figure 5e,f).

To evaluate whether downregulation of the PI3K/AKT pathway could affect TORC1 activity, we analyzed the protein expression of the downstream effector of TORC1, p70 S6K, phosphorylated at Thr389. In the U251 cell line, both 100 μM APE and N8 (100 μM and 25 μM) caused a decrease in the expression of Phospho-p70 S6K (Thr389) compared with the unphosphorylated form (Figure 5g). As shown in Figure 5h, a reduction in the Phospho-p70 S6K (Thr389) expression was observed in GB7 cells treated with high- or low-dose N8, whereas no significant change was observed after 72 h of APE treatment compared with untreated cells.

Another factor involved in the regulation of TORC1 is AMPK, which inhibits the activity of this complex. Western blot analysis showed that M2 mAChR activation with both agonists caused increased protein expression of Phospho-AMPKα (phosphorylated at Thr172), compared with inactive AMPKα, in the U251 cell line (Figure 5i). In GB7 cells, we evidenced the same trend only after N8 treatments, whereas in the APE-treated cells, the active AMPKα form was not significantly modulated with respect to the control condition (Figure 5j).

### 3.3. Analysis of Apoptosis Induction Following M2 mAChR Activation

Since an excessive level of autophagy could promote cell death, including the apoptotic process [42], we evaluated the expression of proteins that are known to be activated during apoptosis, i.e., Caspase-9 and Caspase-3, also prolonging the treatment with M2 agonists applied in the autophagy experimental protocol for an additional 24 h.

**Figure 6:**
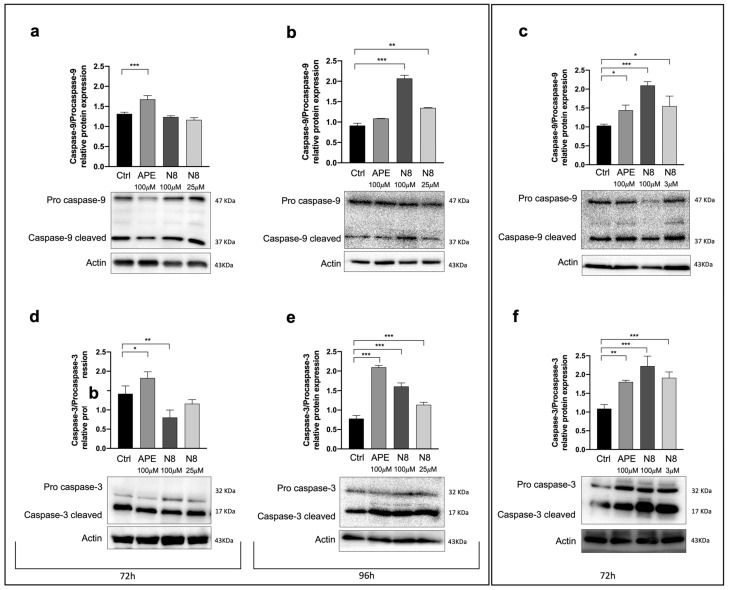
Representative Western blot analysis of Caspase-9 cleaved/Procaspase-9 expression after 72 h of treatment (**a**) and after 96 h of treatment (**b**) with 100 μM APE, 100 μM N8 or 25 μM N8 in the U251 cell line. (**c**) Representative Western blot analysis of Caspase-9 cleaved/Procaspase-9 expression after 72 h of treatment with 100 μM APE, 100 μM N8 or 3 μM N8 in GB7 cells. The graphs show the densitometric analysis of the bands of Western blot analysis for Caspase-9 cleaved normalized with the bands of Procaspase-9 protein. Actin was used as an internal reference protein. Representative Western blot analysis of Caspase-3 cleaved/Procaspase-3 expression after 72 h of treatment (**d**) and after 96 h of treatment (**e**) with 100 μM APE, 100 μM N8 or 25 μM N8 in the U251 cell line. (**f**) Western blot analysis of Caspase-3 cleaved/Procaspase-3 expression after 72 h of treatment with 100 μM APE, 100 μM N8 or 3 μM N8 in GB7 cells. The graphs show the densitometric analysis of the bands of Western blot analysis for Caspase-3 cleaved normalized with the bands of Procaspase-3 protein. Actin was used as an internal reference protein. The data are the average (mean ± SEM) of three independent experiments. The ANOVA test was used, followed by Dunnett’s post-test to identify the statistical significance between pairs, untreated cells (Ctrl) vs. M2 agonist-treated cells (*** *p* < 0.001, ** *p* < 0.01, * *p* < 0.05). The uncropped blots are shown in Supplementary Materials.

For the U251 cell line, we analyzed the expression of Caspase-9 and Caspase-3 proteins after 72 h and 96 h of treatment. As shown in Figure 6a, after 72 h of 100 μM APE treatment, the Caspase-9 cleaved/Procaspase-9 ratio increased compared with that for untreated cells (Ctrl). In contrast, no significant modulation was observed in cells treated with a high dose (100 μM) or a low dose (25 μM) of N8. The same trend was monitored for Caspase-3 after 72 h of treatment with the M2 receptor agonists (Figure 6d). After 96 h of treatment, we detected an increased expression of the cleaved forms of Caspase-9 (Figure 6b) and Caspase-3 (Figure 6e) with 100 and 25 μM N8 doses. Similarly, after 72 h treatment with the M2 agonists (100 μM APE, 100 and 3 μM N8), in GB7 cells, the active forms of Caspase-9 (Figure 6c) and Caspase-3 (Figure 6f) showed an enhanced expression compared with the control cells.

**Figure 7:**
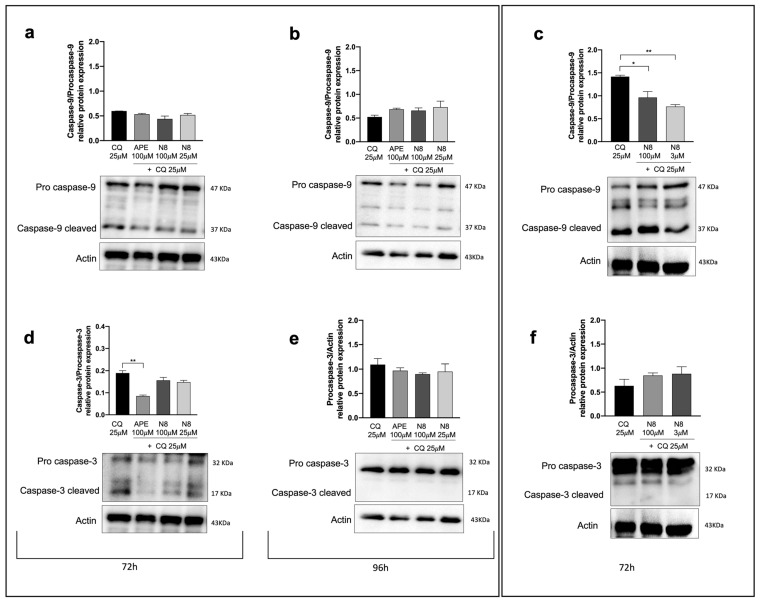
Representative Western blot analysis of Caspase-9 cleaved/Procaspase-9 expression after 72 h of treatment (**a**) and after 96 h of treatment (**b**) with 25 μM CQ, 100 μM APE + 25 μM CQ, 100 μM N8 + 25 μM CQ or 25 μM N8 + 25 μM CQ in the U251 cell line. (**c**) Representative Western blot analysis of Caspase-9 cleaved/Procaspase-9 expression after 72 h of treatment with 25 μM CQ, 100 μM N8 + 25 μM CQ or 3 μM N8 + 25 μM CQ in GB7 cells. Graphs show the densitometric analysis of the bands of Western blot analysis for Caspase-9 cleaved normalized with the bands of Procaspase-9 protein. Actin was used as the internal reference protein. (**d**) Representative Western blot analysis of Caspase-3 cleaved/Procaspase-3 expression after 72 h of treatment. The graph shows the densitometric analysis of the bands of Western blot analysis for Caspase-3 cleaved normalized with the bands of Procaspase-3 protein. Representative Western blot analysis of Caspase-3 cleaved/Procaspase-3 expression (**e**) after 96 h of treatment with 25 μM CQ, 100 μM APE + 25 μM CQ, 100 μM N8 + 25 μM CQ or 25 μM N8 + 25 μM CQ in the U251 cell line and (**f**) after 72 h of treatment with 25 μM CQ, 100 μM N8 + 25 μM CQ or 3 μM N8 + 25 μM CQ in GB7 cells. Graphs show the densitometric analysis of the bands of Western blot analysis for Procaspase-3 normalized with the bands of actin protein. Actin was used as the internal reference protein. (**e**,**f**) No statistical differences were observed between all experimental conditions (n.s. *p* > 0.05). Data are the average (mean ± SEM) of three independent experiments. The ANOVA test was used to evaluate the statistical significance of group differences, followed by Dunnett’s Multiple Comparison Test to identify the statistical significance between pairs, Ctrl (untreated cells) vs. M2 agonist-treated cells (** *p* < 0.01, * *p* < 0.05). The uncropped blots are shown in Supplementary Materials.

To evaluate whether the activation of the apoptotic process is a consequence of M2 receptor-induced autophagy, Western blot analysis for Caspase-9 and Caspase-3 was performed in both cell lines in the presence of CQ, either alone or in combination with the M2 agonists. Figure 7a,d showed that after 72 h of treatment in the U251 cells, the M2 agonist + CQ did not cause an increase in the cleaved form of both Caspases 3 and 9, at variance with what we had observed in cells treated with the M2 agonist alone (Figure 6a,d). The same trend was detected for Caspase-9 in the U251 cells, after 96 h of treatment (Figure 7b), while the cleaved form of Caspase-3 was completely absent under all experimental conditions (Figure 7e). In GB7 cells, the expression of caspases after 100 μM APE + CQ treatment was not evaluated, because the same concentration of APE alone did not cause an increase in autophagy (see Figure 4e). Also, in these cells, the presence of CQ in combination with N8 both at high (100 μM) and low (3 μM) doses did not induce the expression of the cleaved form of Caspase-9, which in this case is reduced with respect to the control with CQ alone (Figure 7c). Like in U251 cells after 96 h of treatment, in GB7 cells, we did not detect the cleaved form of Caspase-3 but found a strong accumulation of Procaspase-3 in all experimental conditions (Figure 7f).

## 4. Discussion

Over the past decade, various studies have shown that the activation of mAChRs is critical in the regulation of cell proliferation and cancer progression [43]. In this context, we have been studying the role of the M2 mAChR in cancer for many years, demonstrating that activation of this receptor subtype brings about anti-proliferative and cytotoxic effects on cells of different tumor types such as ovarian cancer [19], breast cancer [18], neuroblastoma [21] and GBM [32]. For GBM, we showed that in the U251 cell line, the activation of M2 mAChRs by the orthosteric agonist APE caused cell cycle arrest as well as progressive accumulation of the cells in the G2/M phase [24]. In GSCs cells, named GB7, APE and the dualsteric agonist N8 were capable of inhibiting cell proliferation in a time- and dose-dependent manner [23,33]. Moreover, analysis of the U251 cell fraction with hypodiploid DNA content and higher granularity (SSC) obtained by flow cytometry, and the ELISA quantification of cytoplasmic nucleosomes, showed that treatment with 100 μM APE for 72 h caused an increase in apoptotic cells [24]. Once the same analysis was performed in GB7 cells after 48 h and 72 h treatment with 100 μM N8, this M2 mAChR activator produced an enhanced percentage of apoptotic cells [33].

At present, the role of autophagy in cancer cells is receiving much attention since this process seems to have divergent effects depending on tumor type and/or grade. Indeed, it has been observed that autophagy plays a strategic role in the regulation of tumor progression, acting as a tumor suppressor in the early stages of tumor development and as a pro-survival mechanism in the later stages of tumorigenesis [1]. A recent study has shown that combined treatment with temozolomide and rapamycin, an mTOR pathway inhibitor, causes overexpression of Beclin-1 and LC3-II, thus considerably increasing autophagy-induced cell death of U251 cells [44]. Building on this evidence, by transfection of constructs overexpressing LC3B-EGFP, we demonstrated that the autophagic process is promoted after activation of M2 mAChRs in the U251 stable cell line by both M2 orthosteric (APE) and dualsteric (N8) agonists (Figure 2). With a pH-sensitive expression construct, mRFP-EGFP-LC3B, we demonstrated that M2 agonists upregulate autophagy without causing its arrest. In fact, the increase in LC3 aggregates was observed only after the addition of CQ, an autophagy flux blocker, and no summative effect between CQ and the applied M2 agonist was put in evidence (Figure 3). The ability of M2 mAChR activation to promote autophagy was also confirmed by the analysis of LC3B protein expression by Western blotting (Figure 4). In this experiment, human GSCs were also assayed, to compare the effects of M2 agonists on a human established cell line (U251 cell line) with those on human GSCs (GB7 cells). Western blot analysis confirmed that N8 was able to induce autophagy both in the U251 cell line and GB7 cells, while APE was able to increase LC3B-II compared with LC3B-I only in the U251 cell line. The combined treatment with M2 agonists, which alone caused an increase in LC3B-II expression, and CQ significantly increased the LC3B-II/LC3B-I ratio when compared with the treatment with CQ alone (Figure 4d,f). These results, together with those from the cells transfected with LC3-EGFP and mRFP-GFP-LC3B vectors, confirm the ability of M2 agonists to promote autophagic flux and allow the fusion between autophagosomes and lysosomes; as a matter of fact, only CQ blocked the autophagy process at the autophagosome stage. However, a difference between the two studied M2 agonists clearly emerged since APE was found to induce autophagy in U251 cells only, whereas N8 was efficacious in both U251 and GB7 cells.

**Figure 8:**
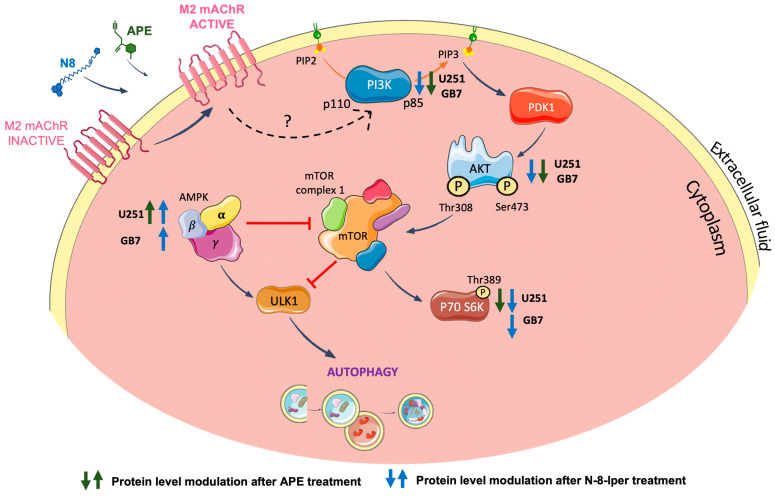
Schematic representation of the modulation of PI3K/AKT/TORC1 signaling pathway and AMPK activity downstream of M2 mAChR activation by APE or N8 in U251 and GB7 cell lines. Green and blue arrows indicate the modulation of the corresponding protein after treatment with APE and N8, respectively.

To further explore the different patterns of autophagy modulation by the two M2 agonists in both cell lines, we analyzed the expression of TORC1 and AMPK, two main factors involved in the regulation of this process. In both cell lines, treatment with N8 for 72 h, at high and low doses, downregulated the protein expression of PI3K p85 (Figure 5a–c); the active form of AKT (phosphorylated at Thr308) (Figure 5d–f); and the active form of the downstream effector of TORC1, p70 S6K phosphorylated at Thr389 (Figure 5g–h). APE produced the same downstream effects but only in U251 cells, while in GB7 cells, it negatively modulated the PI3K/AKT pathway but did not cause significant downregulation of the active form of p70 S6K, indicating a failure in TORC1 downregulation. Regarding the AMPKα protein level, an increase in the phosphorylated (active) form over the non-phosphorylated (inactive) one was observed after treatment with both APE and N8, at high and low doses (Figure 5i). In GB7 cells, only N8 was able to significantly raise the Phospho-AMPKα protein levels, further confirming the inability of APE to promote autophagy in GSCs (Figure 5j). In Figure 8, we summarize the signaling pathways of the M2 receptor-induced autophagy process emerging from the above-discussed data. It is worth highlighting the upregulation of AMPKα and downregulation of TORC1 activity following treatment with the dualsteric muscarinic agonist N8 in both cell lines. Conversely, the orthosteric agonist APE engendered a parallel profile of autophagy flux modulation only in U251 cells and not in GB7 cells.

As already underlined, several literature data suggest that autophagic flux may lead to cell death, including apoptosis [42,45]. In previous investigations, we demonstrated that the two M2 agonists induce cytotoxic effects [24,33], and using Fluorescence-Activated Cell Sorting (FACS) analysis, we found that they caused cell cycle arrest and apoptotic cell death [24,33,34]. To further support these results and delve into the correlation between apoptotic cell death and M2 receptor-induced autophagy, we analyzed the expression of two of the proteins involved in the apoptotic process, i.e., Caspase-9 and Caspase-3 (Figure 6). A treatment of 72 h with APE in U251 cells induced an increase in the cleaved forms of both Caspase-9 (Figure 6a) and Caspase-3 (Figure 6d), confirming our previous data. It was necessary to extend the treatment to 96 h to observe the increase in these proteins in N8-treated U251 cells (Figure 6b,e). In GB7 cells, the cleaved forms of Caspases 9 (Figure 6c) and 3 (Figure 6f) were upregulated by N8. The treatment duration is indicative of a direct sequence between autophagy and apoptotic events. Unlike N8 treatment, the upregulation of apoptotic proteins after APE treatment does not seem to be linked with autophagy in GB7 cells.

Treatment with the autophagy inhibitor CQ allowed for an improvement in the correlation between autophagy and downstream apoptosis. In fact, the inhibition of autophagic flux by CQ caused a remarkable reduction in or complete absence of caspases in their active form, differently from what we observed when treating cells with N8 or APE alone. These results suggest that the prevention of the autophagy process, which is promoted by M2 agonists (N8 for the U251 cell line and GB7 cells, APE for U251 cells only), interferes with the activation of apoptosis.

According to previous data, both APE and N8 have anti-proliferative effects, interfering with PI3K/AKT pathway, in both cell models [23,33]. Downregulation of the PI3K/AKT/TORC1 pathway, involved in regulating the proliferation and activation of autophagy, can lead to cell death by apoptosis in both GBM cell models after N8 treatments. Downregulation of the PI3K/AKT pathway after APE treatment in GB7 cells can explain the decreased cell proliferation previously described [34], but it is not able to inhibit TORC1 action, as also suggested by the lack of increased AMPK expression. This evidence explains the lack of upregulation of the autophagic process in GB7 cells after APE treatment. Western blot analysis of caspases and previous results from APE-treated GB7 cells, in which an increased percentage of annexin V-positive cells was assessed [34], suggest that 100 μM APE directly induces apoptosis without any correlation with autophagy [34]. This different APE behavior in GB7 cells compared with U251 may in part be dependent on the genetic background of the two cell lines. GB7 cells present wild-type p53, whereas U251 cells present mutated p53. Considering the anti-proliferative and the strong cytotoxic effects produced by APE in cells where p53 is active, according to its function as a genome guardian and regulator of the cell cycle, it may directly drive the cells to apoptosis.

Differently, N8 and APE seem to trace the activation of the same pathway, inducing autophagy followed by the apoptotic process, in U251 cells, but in GB7 cells the same effects were evident only with N8-Iper.

## 5. Conclusions

The results of this study confirm that selective M2 agonists may counteract cell survival in GSCs and stable GBM cell lines. Indeed, both N8 and APE were found to activate the autophagic process followed by apoptosis by downregulating the PI3K/AKT/TORC1 pathway and upregulating AMPK protein expression. However, in GSCs, APE induces apoptosis but without promoting the autophagic process, and it probably triggers alternative mechanisms that could be mediated by the p53 protein [24,25].

At present, we are unable to speculate on the different activity profiles characterizing the two types of muscarinic agonists, which could be related to some extent to a difference in the activation mode of the M2 mAChR. However, our overall data confirm the promising role of this receptor subtype as a strategic therapeutic target for GBM therapy. Although further analyses are necessary, the ability of dualsteric agonists like N8 to act at low concentrations and activate similar effects in stable and primary GBM cells highlights their relevance in view of a putative therapeutic perspective.

## Data Availability

Data are contained within the article.

## Supplementary Materials

The following supporting information can be downloaded at: https://www.mdpi.com/article/10.3390/cancers16010025/s1, Figure S1: Representative images of fluorescence microscopy analysis of the U251 cell line transfected with the mRFP-GFP-LC3B expression vector, treated for 72 h with 100 μM APE, 100 μM N8 or 25 μM N8 in absence (a) or presence of 25 μM CQ (b). Scale bar = 10 μm.
