# Supplementary material for: M2 Muscarinic Receptor Stimulation Induces Autophagy in Human Glioblastoma Cancer Stem Cells via mTOR Complex-1 Inhibition
Source: Cancers (Basel). 2023 Dec 20;16(1):25. doi: 10.3390/cancers16010025 (PMC10778261; doi:10.3390/cancers16010025)
Supplement: Supplementary file 1 [file cancers-16-00025-s001.zip › cancers-2716493-supplementary.pdf]

cancers

MDPI

Supplementary Materials:

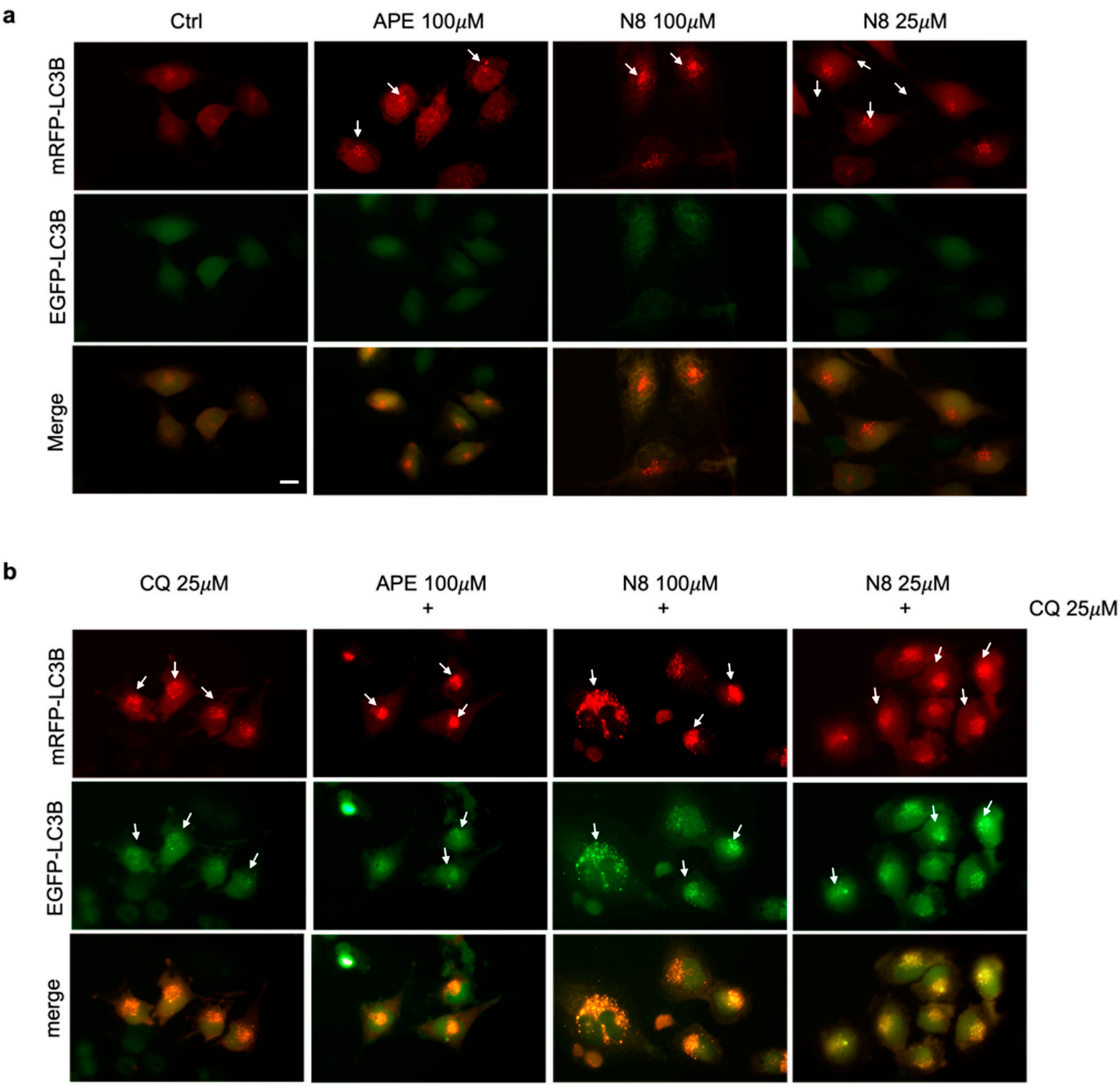

Figure S1. Representative images of fluorescence microscopy analysis of the U251 cell line transfected with the mRFP-GFP-LC3B expression vector, treated for 72 h with 100 µM APE, 100 µM N8 or 25 µM N8 in absent **(a)** or presence of 25 µM CQ **(b)**. Scale bar=10 µm.
